# Inertial-Assisted Immunomagnetic Bioplatform towards Efficient Enrichment of Circulating Tumor Cells

**DOI:** 10.3390/bios11060183

**Published:** 2021-06-05

**Authors:** Yixing Gou, Jiawen Liu, Changku Sun, Peng Wang, Zheng You, Dahai Ren

**Affiliations:** 1 State Key Laboratory of Precision Measurement Technology and Instruments, Tianjin University, Tianjin 300072, China; gouyx@tju.edu.cn (Y.G.); sunck@tju.edu.cn (C.S.); wang_peng@tju.edu.cn (P.W.); 2 State Key Laboratory of Precision Measurement Technology and Instruments, Department of Precision Instrument, Tsinghua University, Beijing 100084, China; liujiawen16@gmail.com (J.L.); yz-dpi@mail.tsinghua.edu.cn (Z.Y.)

**Keywords:** inertial microfluidics, immunomagnetic beads, circulating tumor cells

## Abstract

Serving as an effective biomarker in liquid biopsy, circulating tumor cells (CTCs) can provide an accessible source for cancer biology study. For the in-depth evaluation of CTCs in cancer analysis, their efficient enrichment is essential, owing to their low abundance in peripheral blood. In this paper, self-assembled immunomagnetic beads were developed to isolate CTCs from the ordered bundles of cells under the assistance of the spiral inertial effect. Parametric numerical simulations were performed to explore the velocity distribution in the cross section. Based on this chip, rare CTCs could be recovered under the throughput of 500 μL/min, making this device a valuable supplement in cancer analysis, diagnostics, and therapeutics.

## 1. Introduction

Metastasis is regarded as the primary cause of the high mortality rate among cancer patients. According to the World Health Organization (WHO), at least 30% of these deaths are preventable if early primary tumors are treated before their metastases [1]. Circulating tumor cells (CTCs), which are usually detached from tumor lesions and flow into peripheral blood, can lead to 90% tumor metastases [2], making it a significant biomarker in liquid biopsy. Through the detection of CTCs, we can monitor the dynamic states of tumors [3], evaluate the therapeutic results in real time, prepare personalized medication [4], and provide nucleic acid analysis for precise medical treatment [5]. Nevertheless, the potential of CTCs in clinical medicine has not been fully exploited due to their extremely low concentrations (10–100 CTCs in 10^9^ blood cells) in the peripheral blood of cancer patients [6]. Thus, accurate CTC separation from the blood cells is essential for further cancer diagnosis and medical treatment [7].

In recent years, significant progress has been made in the enrichment of CTCs, based on the differences between blood cells and CTCs in physical structures and immunological characteristics [8,9,10], some of which has been commercialized. The CellSearch system from Veridex, which was the only one approved by the Food and Drug Administration of the United States, utilizes immunomagnetic beads coupled with antibodies to identify and isolate CTCs under the effect of external magnetic fields [11]. Some biomedical companies have also developed products, such as AdnaTest from AdnaGen [12], MACS from Miltenyi [13], and MagSweeper from Illumina [14]. These isolation platforms are all based on immunomagnetic beads, demonstrating their significant potential in clinical and scientific research for the inherent ability of magnets in noncontact manipulation.

In addition to commercial companies, there are several research groups working on integrated CTC isolation microfluidic systems [15,16,17]. Toner’s group presented a highly integrated system combining deterministic lateral displacement (DLD), sinusoidal inertial focusing, and immunomagnetic separation [18], but the low throughput and difficult fabrication limit its further commercialization. Another negative CTC enrichment system [19,20] has been reported using magnetic beads coated with anti-CD45 antibodies to specifically recognize white blood cells, realizing the label-free isolation of CTCs. However, there would be a loss of rare CTCs due to the complicated processing, including cascade filters. As an emerging but expanding field, inertial microfluidics has increasingly attracted interest owing to its superiority in its simple structure and fabrication, high-throughput process, label-free method, and ease of integration with other methods [7,21]. Based on hydrodynamics, this method would benefit from a laminar flow regime, making it a popular preconcentration process for integrated CTC enrichment systems [22,23].

**Figure 1:**
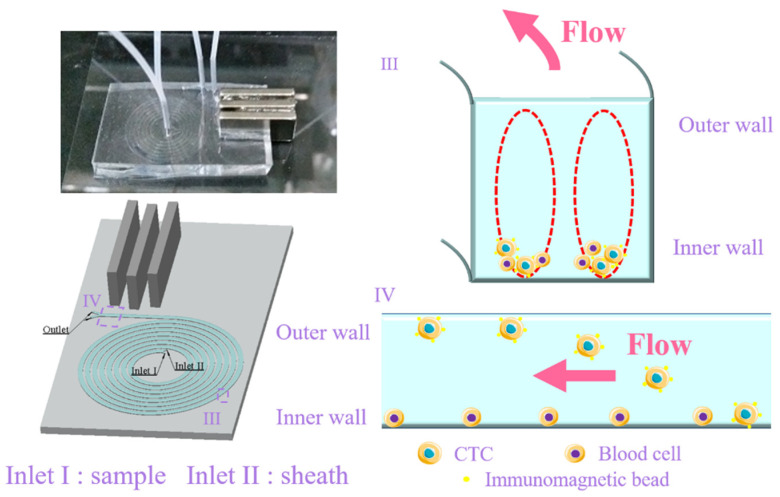
Schematic of the integrated separation biochip. The CTC bonding with immunomagnetic beads were collected from the outer side of the outlet using permanent magnets, and the unbonded blood cells without were collected from the inner side of the outlet with the assistance of the secondary flow in the cross section. I: sample inlet; II: sheath inlet; III: diagram of the cell focusing in the spiral inertial channel; IV: diagram of the cell separation in magnetic separation channel.

In this paper, we propose an integrated system combining spiral inertial focusing and immunomagnetic separation to isolate CTCs from the blood samples (Figure 1). Inertial focusing was used as a preconcentration to provide a uniform streamline of CTCs and blood cells near the inner wall; the immunomagnetic beads dragged the CTCs towards the outer wall under the effect of a magnetic field. Unlike the traditional magnetic separation straight focusing channel with sheath flow, the topological structure of the spiral channel increases space utilization and accelerates the focusing process, which is favorable to the development of the system miniaturization [24,25]. Meanwhile, since the velocity of the sheath flow in the straight focusing channel was at least 10 times that of the sample to assist the particle focusing, the concentration of the focused cells was extremely low; therefore, centrifugation was necessary before the subsequent analysis, which increased the risk of cell losses. In contrast, the spiral inertial microfluidics could avoid cell losses in the straight focusing channel with sheath flow. In addition, specific antibodies were coated on the magnetic beads in a self-assembled manner, which guaranteed the specific capture of CTCs with high purity. A S-Au bond ensured close contact between CTCs and magnetic beads, avoiding the detachment of the magnetic beads. Rare CTCs could be recovered under the throughput of 500 μL/min, indicating the significant potential of the sample preparation in liquid biopsy.

## 2. Materials and Methods

### 2.1. Device Fabrication

The device we proposed consisted of an 8-loop spiral inertial focusing channel and a straight magnetic separation channel adjacent to neodymium magnets, whose mold was patterned on a silicon wafer using SU-8 negative photoresist (Microchem, TX, USA) and standard photolithography. Degassed prepolymerized polydimethylsiloxane polymer (PDMS, Sylgard 184, Dow Corning, MI, USA) was poured onto the mold and baked in an oven at 65 ℃ for 2 h. After being peeled off the mold and treated with oxygen plasma (Harrick Plasma, Ithaca, NY, USA), the PDMS was bonded to the glass. Finally, the completed chip was heated in the oven to strengthen the bonding effect. The magnets integrated on the chip were fabricated from an alloy of neodymium, iron, and boron.

### 2.2. Synthesization and Modification of the Immunomagnetic Beads

**Figure 2:**
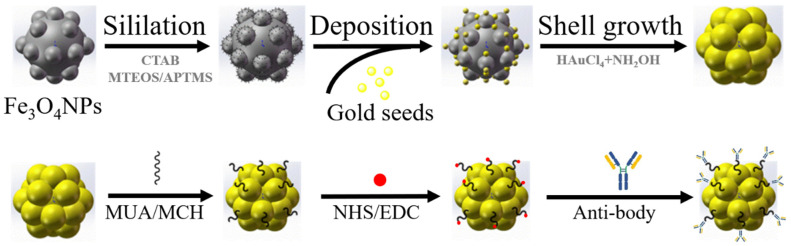
Synthesization and modification process of immunomagnetic beads. The synthesization was based on gold seed growth and sodium citrate reduction methods, and the modification was based on layer-by-layer self-assembly.

The core-shell composite immunomagnetic beads were prepared using the seed-mediated growth [26] and self-assembled method [27], whose synthesization and modification processes are shown in Figure 2. Fe_3_O_4_ magnetic beads (BaseLine, Tianjin, China) were used as the cores, which possessed uniform size, adequate dispersion, and superparamagnetism. First, the magnetic beads were siliated using APTMS to facilitate coupling with gold seed. Subsequently, the gold seeds were deposited on the beads by mixing the gold nanoparticles and magnetic beads and stirring mechanically overnight. Second, formalin solution was added dropwise to the gold-seeded magnetic beads for 30 min, and the mixture was mechanically stirred until the solution changed color from light purple to gray-black. Finally, the gold-coated magnetic beads were synthesized after ultrasonic treatment.

The processed magnetic beads were coated with antibodies using the layer-by-layer self-assembled strategy. First, the magnetic beads were centrifuged and dispersed in ethanol containing 11-Mercaptoundecanoic acid (MUA, Sigma Aldrich, MO, USA) and 6-Mercapto-1-hexanol (MCH, Sigma Aldrich, MO, USA) overnight to form stable Au-S bonds on the gold shell. Subsequently, the external carboxyl group was activated under the effect of N-Hydroxysuccinimide (NHS, Sigma Aldrich, MO, USA) and N-(3-Dimethylaminopropyl)-N′-ethylcarbodiimide (EDC, Sigma Aldrich, MO, USA) dissolved in ultrapurified water (Sartorius arium pro and advance, Göttingen, Germany). Finally, the modified magnetic beads were resuspended in PBS containing anti-EpCAM (Abcam, Cambridge, UK) for at least 1 h. The immunomagnetic beads modified through the layer-by-layer self-assembled method enabled the directional and stable linking due to the strong bond energy of the Au-S bond, avoiding the detachment of the beads.

### 2.3. Cell Culture and Sample Preparation

The breast cancer cells (MCF-7 cell line) used in the experiment were cultured in Dulbecco’s modified Eagle’s medium (DMEM, Invitrogen, CA, USA) supplemented with 10% fetal bovine serum (FBS, Invitrogen, CA, USA) and 1% penicillin-streptomycin (PS, Invitrogen, CA, USA) at 37 °C with 5% CO_2_. Subsequently, the tumor cells were detached from the cell culture flask using trypsinization and resuspended in PBS.

Peripheral blood samples were extracted from donors and stored in a vacuum tube containing K_2_EDTA anticoagulant. Informed consent was obtained from the volunteers at the Peking University People’s Hospital. The number of red blood cells in the whole blood is enormous (~10^9^ cells per mL) compared with the number of white blood cells and CTCs, so it is essential to lyse the red blood cells using the red blood cell lysis buffer. The platelets were removed through centrifugation before the experiment was conducted. The remaining blood cells were spiked with MCF-7 cells for a further separation experiment.

### 2.4. Experimental Setup

In the experiment, two syringe pumps (Harvard Apparatus, MA, USA) were used to inject the cell samples and sheath into the chip within a range of adjustable flow rates. An inverted fluorescence microscope (Olympus IX73, Tokyo, Japan) and an industrial camera (MV-EM120M, MicroVision, xi’an, China) were employed to observe and capture images. The images were subsequently analyzed using the software ImageJ to visualize the capability of multi-particle focusing. Cell counting was conducted using a flow cytometer (BD LSRFortessa, NJ, USA), enabling the identification and distribution analysis of different cells.

## 3. Results and Discussion

### 3.1. Performance Test of Fe_3_O_4_@Au Nanoparticles

**Figure 3:**
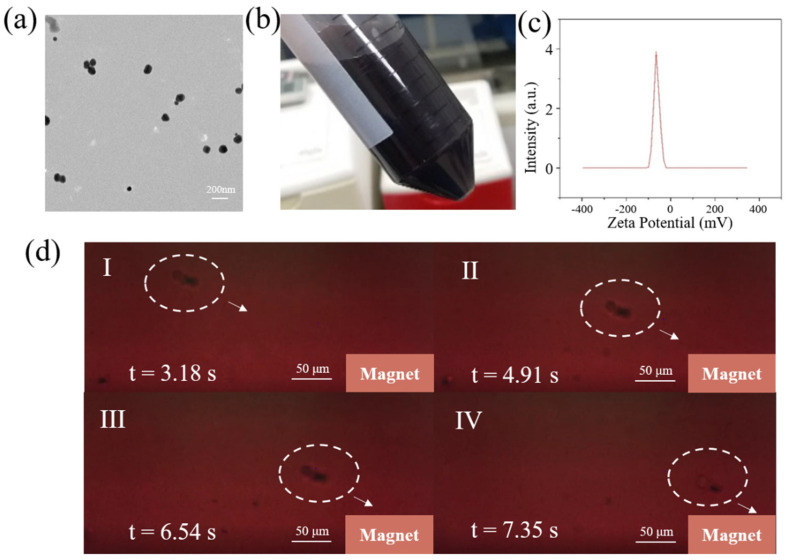
(**a**) The TEM of immunomagnetic beads; (**b**) the solution containing prepared immunomagnetic beads; (**c**) the zeta potential of the beads was measured as −63.0 mV; (**d**) the off-chip pre-experiment in the culture dish, demonstrating that the magnetic force between the immunomagnetic beads and the magnet was strong enough to drag cells and cell clusters to migrate directly.

The Fe_3_O_4_@Au nanoparticles synthesized using gold seed growth and sodium citrate reduction and modified with layer-by-layer self-assembly are characterized in Figure 3a. As the TEM image shows, Fe_3_O_4_@Au exhibited a uniform spherical structure with a diameter of approximately 110 nm. The zeta potential of the solution, which represented the stability behavior of the colloidal dispersion, was measured to be −63 mV (Figure 3c), demonstrating the excellent stability of the solution at the temperature of 25.0 °C. The off-chip pre-experiment was conducted in the culture dish, where a magnet was arranged next to the dish. The continuous captured image shown in Figure 3d suggests that the magnetic force between the immunomagnetic beads and the magnet was strong enough to drag cells, even cell clusters (two or more cells).

### 3.2. Velocity Distribution in the Spiral Inertial Channel

**Figure 4:**
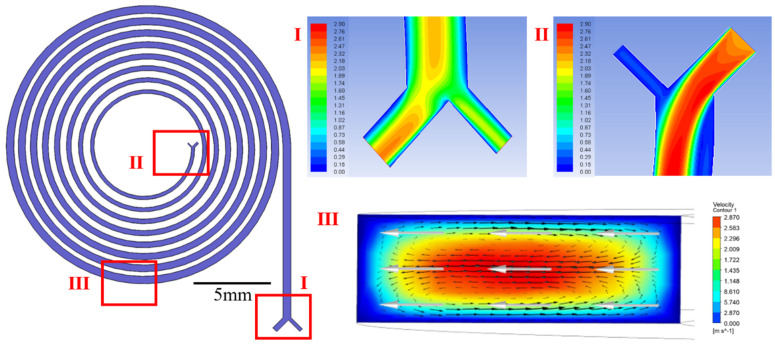
Distribution of flow field in the spiral inertial focusing channel, where the planar simulations of outlet and inlet are indicated by sections I and II. Section III presents the Dean vortex in the cross section of the spiral inertial channel.

Spiral inertial microfluidics is the mainstream structure for particle focusing and separation due to its superior performance from its ultrahigh throughput, smaller size, and faster focusing process [28,29]. After the evaluation of the focusing efficiency, an eight-loop spiral channel was selected as the preconcentration channel. Two inlets were arranged, one with the cell samples and the other with the buffer. Thus, the focusing process could be accelerated considerably, and the rare tumor cell samples could be saved with assistance from the buffer. Numerical simulations were conducted using incompressible Newtonian laminar flow in the computational domain to explore the effect of Dean flow in the cross section under proper initial conditions (velocity inlet and pressure outlet) and boundary conditions (no-slip) [30]. The computational domain was calculated by solving nonsimplified Navier–Stokes (N–S) equations, whose nonlinear inertial term was not negligible. In the simulation, we set the throughput of samples as 500 μL/min and adjusted the throughput of sheath to predict the velocity distribution. The velocity distributions in the outlet, inlet, and cross section are shown in sections I, II and III in Figure 4. Owing to the parabolic velocity profile of Poiseuille flow, the velocity in the central region was higher than that in the wall region; thus, the fluid in the channel flowed laterally from the central region to the wall region [31]. Conversely, the fluid near the wall flowed back from the wall region to the central region to satisfy the mass conservation. As a result, two vortexes (Dean Vortex) with opposite rotating directions were generated in the cross section [32]. The motion of particles was affected by the lateral inertial lift force (F_L_) and Dean drag force (F_D_), and the equilibrium positions of particles were then determined by the interaction of these two forces.

### 3.3. Particle Focusing Characterization in the Spiral Inertial Microchannel

Although the distribution of the velocity in the channel was investigated previously, the particle focusing performance in the experiment was not fully consistent with the numerical simulation and needed to be optimized and evaluated through experimental exploration. In this study, polystyrene microbeads with diameters of 6, 10, and 20 μm were used to mimic the focusing motions of red blood cells, white blood cells, and CTCs, respectively. To explore the optimum focusing condition, we set the velocity of the samples diluted in the PBS as 500 μL/min and adjusted the velocity of the sheath within a range of 750–1500 μL/min. Under different conditions, particle migration was observed, and the focusing positions were recorded using an industrial CCD camera. The focusing positions and width of focused streamlines for each particle were observed by stacking the discrete pictures captured in every shutter and by investigating the intensity profile across the channel width using ImageJ.

**Figure 5:**
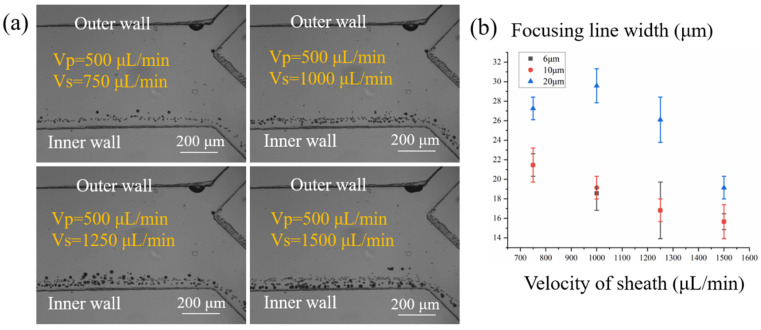
(**a**) Focusing performance of the microspheres with diameters of 6, 10, and 20 μm under a range of velocities; (**b**) the focusing line width was used to evaluate the focusing efficiency of different particles.

The lateral positions of the multi-particle stream under different velocities of the sheath were shown in Figure 5a. When the velocity of the sheath was lower than 1 mL/min, the smaller particles (6 and 10 μm) were located close to the inner wall, while the larger particles (20 μm) were not precisely focused. When the velocity of the sheath was higher than 1 mL/min, the focusing positions of the three particles were all located closer to the inner wall, demonstrating the powerful focusing effect on the particles of the eight-loop spiral channel. The focusing line width was analyzed to quantitatively reveal the focusing performance of different particles (Figure 5b). By investigating the recorded data, we found that the focusing performance could be significantly improved by increasing the velocity of the sheath flow. The width of the focusing line decreased abruptly when the velocity was above 1 mL/min; when the velocity of sheath flow reached 1.5 mL/min, a nearly single-particle train was successfully achieved for three types of particles. In this situation, the throughput of samples could be maintained as 500 μL/min owing to the spiral inertial channel, which is superior to the immunomagnetic separation in straight channel.

### 3.4. Separation Performance of the Integrated Biochip with Inertial Focusing and Immunomagnetic Capture

**Figure 6:**
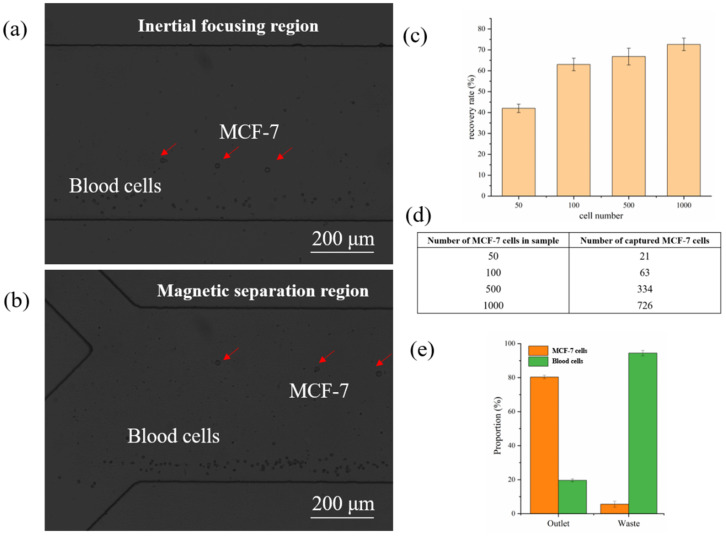
(**a**) The migration performance of the blood cells and MCF-7 cells in the inertial focusing region, where the migrating trajectories of blood cells were located near the inner wall and the MCF-7 cells began to migrate towards the outer wall; (**b**) the migration performance of the blood cells and MCF-7 cells in the magnetic separation region, where MCF-7 cells were dragged by the immunomagnetic beads and flowed out from the separation outlet while the blood cells flowed out from the waste outlet; (**c**) the recovery rate of different numbers of spiked MCF-7 cells; (**d**) the spiked number and captured number of MCF-7 cells, and (**e**) proportion of MCF-7 cells and blood cells in outlet and waste.

The separation experiment was conducted using the lysed peripheral blood spiked with CTCs. Here, we employed MCF-7 cells that overexpressed EpCAM to represent CTCs in our experiment, and the main components of the cell samples were blood cells and MCF-7 cells. Before being spiked with blood cells, the MCF-7 cells were cocultured with Hoechst 33342 for 3 min to dye the cell nucleus for subsequent fluorescence identification with a flow cytometer, and then cocultured with the immunomagnetic beads and gently stirred for 30 min to ensure a dispersed state. The inertial focusing performance of cell samples at the end of the spiral channel was shown in Figure 6a. In the inertial focusing region, the migrating trajectories of blood cells were similar to the polystyrene microbeads and located near the inner wall. The MCF-7 cells, whose migrating performance is slightly different from the polystyrene microbeads, began to migrate towards the outer wall owing to the weak magnetic field. During a long-distance migration in the magnetic separation region, the MCF-7 cells were still dragged by the immunomagnetic beads and kept moving towards the outer wall before flowing out from the separation outlet, which demonstrated the excellent dragging performance of the immunomagnetic beads, while the blood cells were collected from the waste outlet (Figure 6b). The recovery rate of the MCF-7 cells under different spiked cell numbers in 1 mL is shown in Figure 6c, and the proportion of MCF-7 cells and blood cells is summarized in Figure 6e. From these diagrams, we observed that most MCF-7 cells could be recovered after separation with high purity, providing a purified sample for subsequent cancer analysis. Moreover, the proposed positive cell capture strategy could guarantee a higher purity (~80%) than the negative separation strategy, which is favorable for downstream CTC analysis. The blood cells collected in the collection outlet were due to the nonspecific binding of immunomagnetic beads. However, due to the cell heterogeneity, the biomarker EpCAM could not be expressed in all CTCs, which decreased the recovery rate. Therefore, more highly expressed biomarkers should be introduced into this system to further enhance the recovery rate.

## 4. Conclusions

In this paper, we present an integrated biochip combining inertial focusing and immunomagnetic separation for the efficient enrichment of CTCs. The main module of this separation strategy is the immunomagnetic separation, where the immunomagnetic beads are modified through the layer-by-layer self-assembled method, and the spiral inertial channel is used to assist the particle to be focused into a cell bundle. The spiral channel could prevent the cell loss in past magnetic separation studies and increase the space utilization, which is favorable to the system miniaturization. Parametric numerical simulations were performed to determine the velocity distribution in the cross section and guide the velocity range used in the experiment. The eight-loop spiral inertial microfluidic chip was employed to serve as a preconcentration and enable multiple particles to be accumulated into one bundle of cells, which could be used as an on-chip flow cytometer. After the evaluation, rare CTCs could be recovered under the high throughput of 500 μL/min with the assistance of the spiral inertial channel, which reduces the cell processing time. Compared with passive separation methods without any cell binding, higher purity (~80%) could be achieved using the specific cell capture strategy, which is favorable for the following molecular analysis. Since EpCAM could not be expressed in any CTCs, more highly expressed biomarkers could be introduced to further improve the recovery rate, as well as the specificity. This immunomagnetic strategy can also be applied to the CTCs that do not express EpCAM, which could be identified through their unique biomarkers binding with magnetic beads. This separation system can be a valuable tool in cancer analysis, diagnostics, therapeutics, and related areas.
